# Glucagon-like peptide-1 receptor agonist versus other anti-diabetic agents on patients with prostate cancer undergoing androgen-deprivation therapy

**DOI:** 10.3389/fruro.2026.1855180

**Published:** 2026-08-07

**Authors:** Sheng-Chi Huang, Wan-Yu Cheng, Cheng-You Hou, Jheng-Yan Wu, Chih-Cheng Lai, Wen-Hsin Tseng

**Affiliations:** 1 Department of General Medicine, Chi Mei Medical Center, Tainan, Taiwan; 2 Division of Urology, Department of Surgery, Chi Mei Medical Center, Tainan, Taiwan; 3 Department of General Medicine, Taipei Medical University Hospital, Taipei, Taiwan; 4 Department of Nutrition, Chi Mei Medical Center, Tainan, Taiwan; 5 Department of Public Health, College of Medicine, National Cheng Kung University, Tainan, Taiwan; 6 Department of Intensive Care Medicine, Chi Mei Medical Center, Tainan, Taiwan; 7 School of Medicine, College of Medicine, National Sun Yat-sen University, Kaohsiung, Taiwan

**Keywords:** androgen deprivation therapy, DPP-4I, GLP-1 receptor agonist, mortality, prostate cancer, SGLT2 inhibitor

## Abstract

Patients with prostate cancer (PCa) undergoing androgen deprivation therapy (ADT) frequently develop cardiometabolic complications, particularly those with type 2 diabetes (T2D). Glucagon-like peptide-1 receptor agonists (GLP-1RAs) provide cardiovascular and renal benefits in diabetic populations, but comparative evidence with other glucose-lowering therapies in men receiving ADT remains limited. We conducted a retrospective cohort study using the TriNetX network. Adults with T2D and PCa undergoing ADT who received GLP-1RA, dipeptidyl peptidase-4 inhibitors (DPP-4is), or sodium–glucose cotransporter-2 inhibitors (SGLT2is) between January 2005 and December 2025 were included. Propensity score matching generated balanced cohorts for two comparisons: GLP-1RA versus DPP-4i and GLP-1RA versus SGLT2i. The primary outcome was all-cause mortality; secondary outcomes were major adverse cardiovascular events (MACEs), major adverse kidney events (MAKEs), and thrombotic events. After matching, 659 patients per group were included in the GLP-1RA versus DPP-4i comparison and 1,009 per group in the GLP-1RA versus SGLT2i comparison. Compared with DPP-4i, GLP-1RA use was associated with lower all-cause mortality (HR, 0.60; 95% CI, 0.46–0.79), MAKEs (HR, 0.63; 95% CI, 0.48–0.81), and thrombotic events (HR, 0.53; 95% CI, 0.32–0.89), whereas MACEs were similar (HR, 0.87; 95% CI, 0.63–1.12). Compared with SGLT2i, GLP-1RA use was associated with lower all-cause mortality (HR, 0.76; 95% CI, 0.59–0.99), whereas no significant differences were observed for MACEs, MAKEs, or thrombotic events. These neutral findings should be interpreted cautiously because the limited number of events may have reduced statistical power to detect modest between-group differences. In conclusion, among men with PCa receiving ADT and comorbid T2D, GLP-1RA use was associated with lower all-cause mortality than both DPP-4i and SGLT2i, with additional reductions in kidney and thrombotic events versus DPP-4i. GLP-1RAs may represent a favorable therapeutic option in this metabolically and vascularly vulnerable population. Prospective studies are warranted to confirm these associations and clarify the underlying mechanisms.

## Introduction

Prostate cancer is the second most commonly diagnosed malignancy in men worldwide and remains a leading cause of cancer-related mortality. Recent global estimates indicate that it accounts for approximately 7% of incident cancers and nearly 4% of cancer deaths among men (1, 2). With population aging, its burden continues to increase.

Androgen deprivation therapy (ADT) is a cornerstone treatment for advanced and high-risk prostate cancer (1, 3). By suppressing hypothalamic–pituitary–gonadal signaling and reducing circulating testosterone levels, ADT inhibits androgen receptor–dependent tumor growth (4, 5). Current guidelines, such as those from the NCCN, recommend maintaining testosterone at castrate levels to optimize oncologic outcomes (3).

Despite its therapeutic efficacy, ADT is consistently associated with increased cardiometabolic risk. Large observational studies and scientific statements from the American Heart Association have demonstrated higher incidences of diabetes, coronary artery disease, myocardial infarction, and cardiovascular mortality among ADT users (6–8). Cardiovascular and metabolic comorbidities are important contributors to overall mortality in this population.

Furthermore, diabetes mellitus independently predicts worse overall survival and cancer-specific survival in men with prostate cancer (9–11). Therefore, the selection of glucose-lowering therapy in patients receiving ADT may have implications beyond glycemic control, profoundly affecting their overall prognosis.

Recently, glucagon-like peptide-1 receptor agonists (GLP-1RAs) have demonstrated significant reductions in all-cause mortality and major adverse cardiovascular events (MACE) in multiple cardiovascular outcome trials and meta-analyses (12–15). Similarly, sodium-glucose cotransporter-2 (SGLT2) inhibitors have shown profound cardiovascular and renal benefits, improving survival in high-risk populations (16–18). In contrast, dipeptidyl peptidase-4 (DPP-4) inhibitors have generally demonstrated neutral effects on cardiovascular and mortality outcomes (19–22). Increasing real-world and comparative effectiveness evidence supports the cardiovascular advantages of these newer glucose-lowering agents (23–25).

However, comparative effectiveness data evaluating overall survival among these glucose-lowering strategies in men with prostate cancer undergoing ADT remain highly limited. To our knowledge, no large-scale real-world study has directly compared GLP-1RAs, SGLT2 inhibitors, and DPP-4 inhibitors with respect to overall survival in this high-risk population. We therefore conducted a retrospective cohort study using the TriNetX global research network to evaluate the associations of these drug classes with overall survival, alongside secondary analyses of cardiometabolic outcomes in men receiving ADT.

## Methods

### Data source

This retrospective cohort study utilized TriNetX, a global federated health research network that aggregates de-identified electronic health records from approximately 182 million individuals across 157 healthcare organizations (HCOs) (26). The database includes comprehensive clinical information such as diagnostic codes, procedures, prescribed medications, laboratory results, and genomic data. Because only de-identified data were available through the TriNetX platform, investigators had no access to identifiable patient-level information. Institutional review board approval and informed consent were therefore not required.

### Study design

Adults aged ≥18 years with a diagnosis of prostate cancer who were receiving androgen deprivation therapy (ADT) and had pre-existing type 2 diabetes mellitus (T2D) were identified from the TriNetX Research Network between January 1, 2005, and December 31, 2025. Prostate cancer, T2D, ADT exposure, baseline comorbidities, concomitant medications, and study outcomes were identified using standardized ICD-10-CM, ICD-10-PCS, CPT, RxNorm, and other coding systems available within the TriNetX platform. Detailed coding algorithms for all diagnoses, procedures, medications, and outcomes are provided in **Supplementary Table 1**.

ADT exposure was defined as documented receipt of any of the following: (1) surgical castration, identified by CPT codes for radical orchiectomy, simple orchiectomy (with or without testicular prosthesis), or partial orchiectomy, or by the corresponding ICD-10-PCS codes for unilateral or bilateral orchiectomy; (2) gonadotropin-releasing hormone (GnRH) agonists or antagonists, including leuprolide, goserelin, triptorelin, histrelin, degarelix, and relugolix; or (3) androgen receptor pathway inhibitors, including abiraterone, apalutamide, darolutamide, and enzalutamide.

Eligible patients subsequently initiated one of the study glucose-lowering therapies, including glucagon-like peptide-1 receptor agonists (GLP-1RAs), dipeptidyl peptidase-4 inhibitors (DPP-4is), or sodium–glucose cotransporter-2 inhibitors (SGLT2is). The index date was defined as the first recorded prescription date of the initiated study drug after fulfillment of all eligibility criteria.

**Figure 1:**
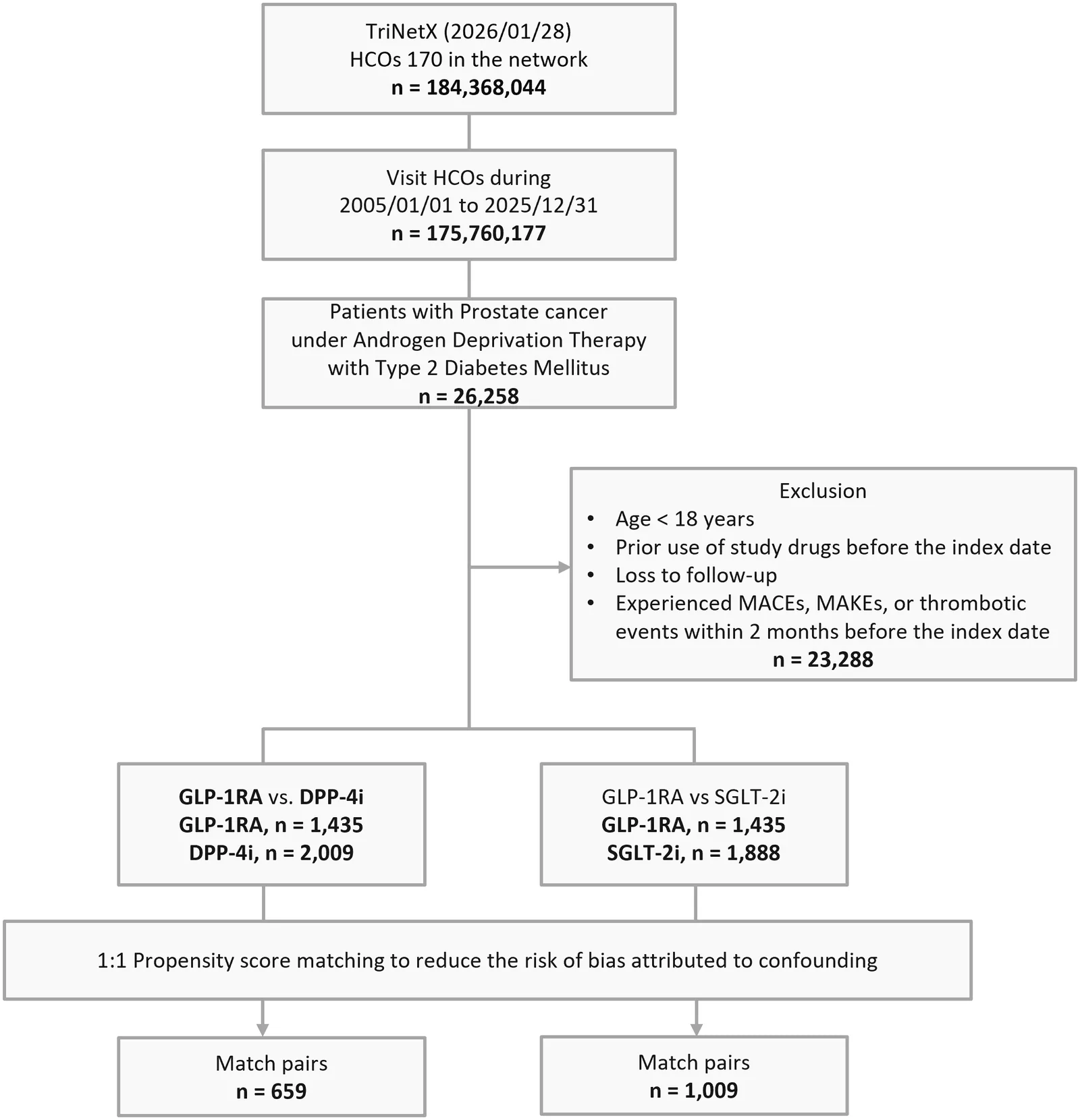
The algorithm for patient selection.

An active-comparator cohort design was employed. Patients receiving GLP-1RAs were compared with those receiving DPP-4is or SGLT2is. Individuals with prior exposure to the index drug class or concurrent use of a comparator drug class before the index date were excluded according to the predefined eligibility criteria to minimize treatment-selection bias. Patients without follow-up after cohort entry or who experienced any study outcome before the start of follow-up were also excluded. Two independent propensity score–matched cohorts were subsequently constructed for the comparisons of GLP-1RA versus DPP-4i and GLP-1RA versus SGLT2i, respectively (**Figure 1**).

### Covariates and propensity score matching

Baseline characteristics were assessed during the 12-month period preceding the index date. Prespecified covariates included demographic characteristics (age, sex, and race/ethnicity), body mass index (BMI ≥30 kg/m²), glycemic control (hemoglobin A1c [HbA1c] ≥9%), renal function (estimated glomerular filtration rate ≤60 mL/min/1.73 m²), and prostate cancer–related laboratory variables (prostate-specific antigen [PSA] ≥0.1 ng/mL and ≥2.0 ng/mL). Baseline comorbidities included chronic kidney disease, atrial fibrillation and flutter, heart failure, cerebrovascular disease, peripheral vascular disease, gout, cirrhosis, other inflammatory liver diseases, hypertension, hyperlipidemia, systemic connective tissue disorders, and type 2 diabetes–related complications. Concomitant medications included lipid-lowering agents, antihypertensives, cardiovascular medications, and glucose-lowering therapies. Detailed definitions and coding algorithms for all baseline covariates are provided in **Supplementary Table 2**.

To reduce measured confounding, propensity scores were estimated using multivariable logistic regression incorporating all prespecified baseline covariates. Patients were matched in a 1:1 ratio using greedy nearest-neighbor matching without replacement with a caliper width of 0.1 standard deviations of the logit of the propensity score. Covariate balance after matching was evaluated using standardized mean differences (SMDs), with an SMD <0.10 considered indicative of adequate balance. Propensity score matching was performed independently for the GLP-1RA versus DPP-4i and GLP-1RA versus SGLT2i comparisons and was repeated separately within each predefined subgroup before estimating treatment effects.

Because detailed oncological characteristics, including tumor stage, Gleason score, metastatic status, castration-resistant disease, and duration of androgen deprivation therapy, were not consistently available as structured variables within the TriNetX database, these variables could not be incorporated into the propensity score model and remain potential sources of residual confounding.

### Outcomes

The primary outcome was all-cause mortality. Secondary outcomes included major adverse cardiovascular events (MACEs), major adverse kidney events (MAKEs), and thrombotic events. MACEs were defined as myocardial infarction, stroke, intracranial hemorrhage, cardiac arrest, or death. MAKEs were defined as stage 5 chronic kidney disease, end-stage renal disease, initiation of dialysis, estimated glomerular filtration rate ≤5 mL/min/1.73 m², or death. Thrombotic events included pulmonary embolism, deep vein thrombosis, and other venous thromboembolic events. Detailed outcome definitions and coding algorithms are provided in **Supplementary Table 3**.

Follow-up began 30 days after the index date (landmark design) to minimize reverse causation and reduce the influence of clinical events occurring immediately after treatment initiation. Patients who experienced the corresponding study outcome before the start of follow-up were excluded from that outcome analysis. Participants were followed until the first occurrence of the outcome of interest, death, the last recorded healthcare encounter, five years after the index date, or the end of available follow-up, whichever occurred first.

### Statistical analysis

Continuous variables are presented as means with standard deviations (SDs), and categorical variables as counts and percentages. Propensity score matching (PSM) was performed before all primary and subgroup analyses to reduce measured confounding and improve comparability between treatment groups. Time-to-event outcomes were analyzed using Cox proportional hazards regression models to estimate hazard ratios (HRs) with corresponding 95% confidence intervals (CIs), fitted separately within each 1:1 propensity score–matched cohort. Kaplan–Meier survival curves were generated to estimate cumulative event incidence, and differences between treatment groups were compared using the log-rank test. The proportional hazards assumption was assessed using the proportionality diagnostics provided by the TriNetX Analytics Platform.

Predefined subgroup analyses were performed for both the GLP-1RA versus DPP-4i and GLP-1RA versus SGLT2i comparisons according to age (≥65 vs. <65 years), obesity, chronic kidney disease, coronary artery disease, and heart failure, as defined at baseline. To preserve covariate balance within each subgroup, separate cohorts were reconstructed within the TriNetX platform, and propensity score matching was repeated independently before estimating treatment effects rather than analyzing subsets of the overall matched cohorts.

To evaluate the robustness of the observed associations to potential residual unmeasured confounding, E-values were calculated for the primary outcome and statistically significant secondary outcomes in each comparison. The E-value represents the minimum strength of association, on the risk-ratio scale, that an unmeasured confounder would need to have with both treatment assignment and the outcome, beyond the measured covariates, to fully explain the observed association (27).

All analyses were performed using the TriNetX Analytics Platform. All statistical tests were two-sided, and a P value <0.05 was considered statistically significant. Because secondary and subgroup analyses were exploratory, these findings should be interpreted cautiously.

## Results

### Patients’ selection

The study utilized the TriNetX global federated health research database, containing 184,368,044 patients as of January 28, 2026. We extracted records for 26,258 patients diagnosed with T2D who had documented healthcare visits. Following the application of specified exclusion criteria, two comparison groups were established to evaluate GLP-1RA against DPP-4i, SGLT2i. To control for potential confounding variables, PSM was employed using a 1:1 ratio for each comparison group. The final matched cohorts consisted of 659 patients per arm for the GLP-1RA versus DPP-4i comparison; 1,009 patients per arm for the GLP-1RA versus SGLT2i comparison (**Figure 1**).

### Baseline characteristics

Before PSM, significant differences in baseline characteristics were found between GLP-1RA users and patients taking DPP-4i, SGLT2i. After PSM, baseline characteristics were well-balanced across most comparison groups, including age, sex, race, BMI, HbA1c levels, comorbidities, T2D-related complications, and medications for cardiovascular disease, hypertension, hyperlipidemia and diabetes management. Baseline balance was achieved in both matched cohorts (GLP-1RA vs. DPP-4i and GLP-1RA vs. SGLT2i), with all standardized mean differences below 0.10 (**Table 1**).

**Table 1:** Post-matched baseline characteristics of glucagon-like peptide-1 receptor agonists (GLP-1RAs) and their comparator cohorts.

	GLP-1RAs	DPP-4i	SMDs	GLP-1RAs	SGLT2i	SMDs
	n=659			n=1,009		
Age at Index (mean ± SD)	76.8 ± 8.3	76.8 ± 8.1	0.003	71.3 ± 7.6	71.4 ± 7.6	0.019
Sex (%)						
Female	0	0	0	0	0	0
Male	659 (100)	659 (100)	0	1,009 (100)	1,009 (100)	0
Ethnicity (%)						
White	393 (59.6)	401 (60.9)	0.025	640 (63.4)	629 (62.3)	0.023
Black or African American	157 (23.8)	151 (22.9)	0.022	247 (24.5)	269 (26.7)	0.05
Asian	30 (4.6)	37 (5.6)	0.048	32 (3.2)	27 (2.7)	0.029
Unknown Ethnicity	142 (21.5)	136 (20.6)	0.022	202 (20)	181 (17.9)	0.053
Race (%)						
Not Hispanic or Latino	460 (69.8)	465 (70.6)	0.017	730 (72.3)	751 (74.4)	0.047
Hispanic or Latino	57 (8.6)	58 (8.8)	0.005	77 (7.6)	77 (7.6)	0
Other Race	30 (4.6)	25 (3.8)	0.038	41 (4.1)	40 (4)	0.005
Unknown Race	44 (6.7)	42 (6.4)	0.012	43 (4.3)	36 (3.6)	0.036
Comorbidities (%)						
Chronic kidney disease	153 (23.2)	149 (22.6)	0.014	233 (23.1)	231 (22.9)	0.005
Atrial fibrillation and flutter	80 (12.1)	71 (10.8)	0.043	136 (13.5)	135 (13.4)	0.003
Heart failure	70 (10.6)	62 (9.4)	0.04	132 (13.1)	131 (13)	0.003
Cerebrovascular diseases	65 (9.9)	55 (8.3)	0.053	82 (8.1)	87 (8.6)	0.018
Peripheral vascular disease	35 (5.3)	35 (5.3)	0	51 (5.1)	55 (5.5)	0.018
Gout	34 (5.2)	36 (5.5)	0.014	51 (5.1)	54 (5.4)	0.013
Cirrhosis	13 (2)	12 (1.8)	0.011	19 (1.9)	13 (1.3)	0.048
Other inflammatory liver diseases	10 (1.5)	10 (1.5)	0	233 (23.1)	231 (22.9)	0.005
Systemic connective tissue disorders	10 (1.5)	10 (1.5)	0	136 (13.5)	135 (13.4)	0.003
Medications (%)						
Antilipemic agents	343 (52)	348 (52.8)	0.015	547 (54.2)	568 (56.3)	0.042
Biguanides	268 (40.7)	281 (42.6)	0.04	407 (40.3)	425 (42.1)	0.036
Antiarrhythmics	239 (36.3)	234 (35.5)	0.016	400 (39.6)	409 (40.5)	0.018
Beta blockers/related	236 (35.8)	224 (34)	0.038	352 (34.9)	372 (36.9)	0.041
Insulins and analogues	224 (34)	219 (33.2)	0.016	349 (34.6)	350 (34.7)	0.002
Diuretics	209 (31.7)	208 (31.6)	0.003	341 (33.8)	337 (33.4)	0.008
Calcium channel blockers	192 (29.1)	185 (28.1)	0.024	304 (30.1)	314 (31.1)	0.022
Ace inhibitors	162 (24.6)	156 (23.7)	0.021	251 (24.9)	248 (24.6)	0.007
Sulfonylureas	136 (20.6)	140 (21.2)	0.015	167 (16.6)	168 (16.7)	0.003
Thiazolidinediones	20 (3)	18 (2.7)	0.018	27 (2.7)	25 (2.5)	0.013
Others (%)						
BMI ≥ 30	323 (49)	325 (49.3)	0.006	597 (59.2)	599 (59.4)	0.004
HbA1c ≥ 9%	144 (21.9)	148 (22.5)	0.015	187 (18.5)	194 (19.2)	0.018
eGFR ≤ 60 (mL/min/1.73 m²)	256 (38.8)	250 (37.9)	0.019	360 (35.7)	376 (37.3)	0.033
PSA ≥0.1 (ng/mL)	295 (44.8)	298 (45.2)	0.009	455 (45.1)	456 (45.2)	0.002
PSA ≥2.0 (ng/mL)	179 (27.2)	187 (28.4)	0.027	251 (24.9)	256 (25.4)	0.011

DPP4i, dipeptidyl peptidase 4 inhibitor; SGLT2i, sodium-glucose cotransporter 2 inhibitor; SMDs, standardized mean difference; SD, standard deviation; BMI, body mass index (kg/m^2^); eGFR, estimated glomerular filtration rate (mL/min/1.73 m²); HbA1c, glycated hemoglobin.(%); PSA, prostate-specific antigen (ng/mL).

### Primary outcome

#### GLP-1RA vs. DPP-4i cohort

**Figure 2:**
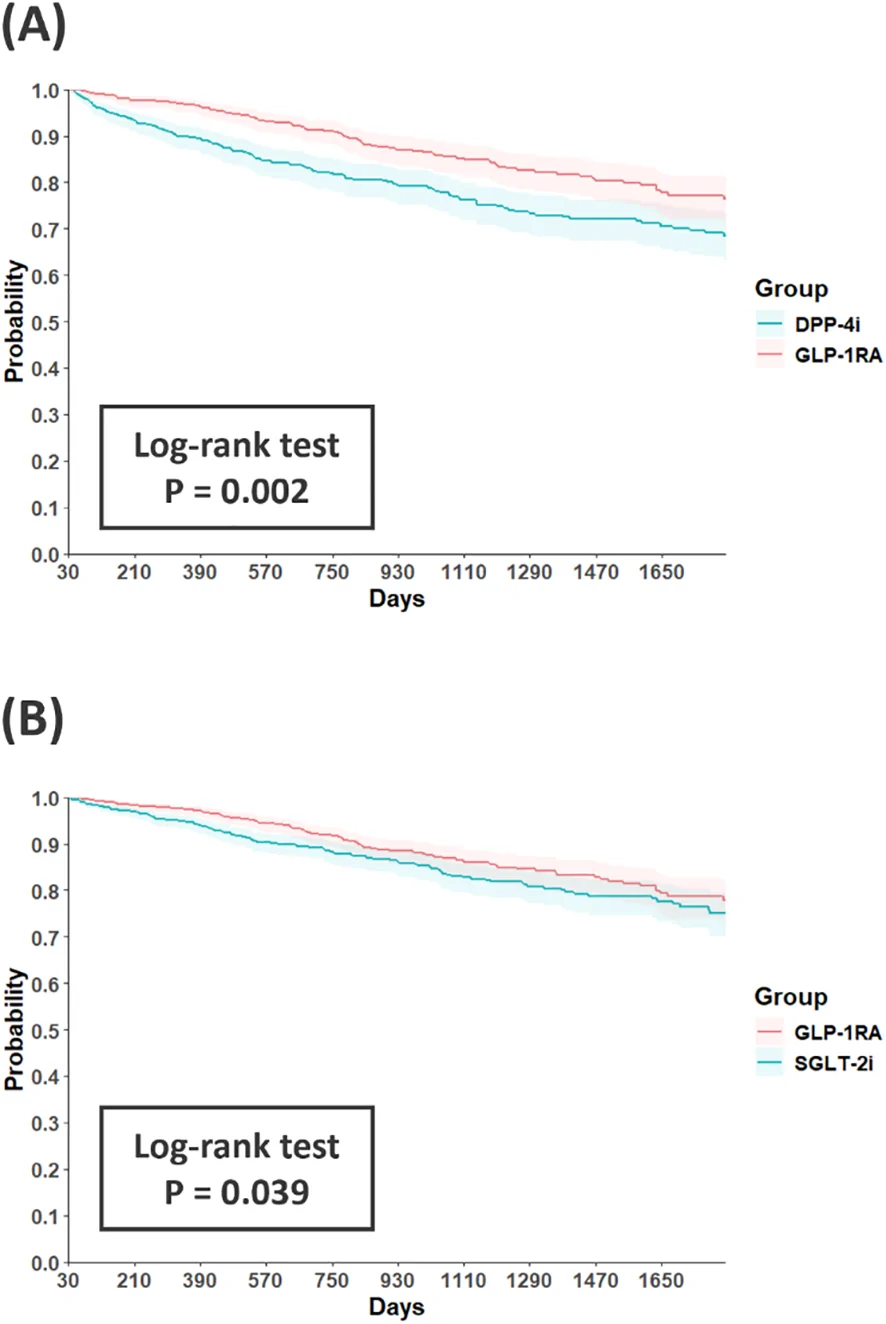
Kaplan-Meier survival curves of patients receiving GLP-1 receptor agonists (GLP-1RA) compared with receiving **(A)**, Dipeptidyl peptidase-4 inhibitors (DPP-4i) **(B)**, SGLT2 inhibitors (SGLT2i).

**Figure 3:**
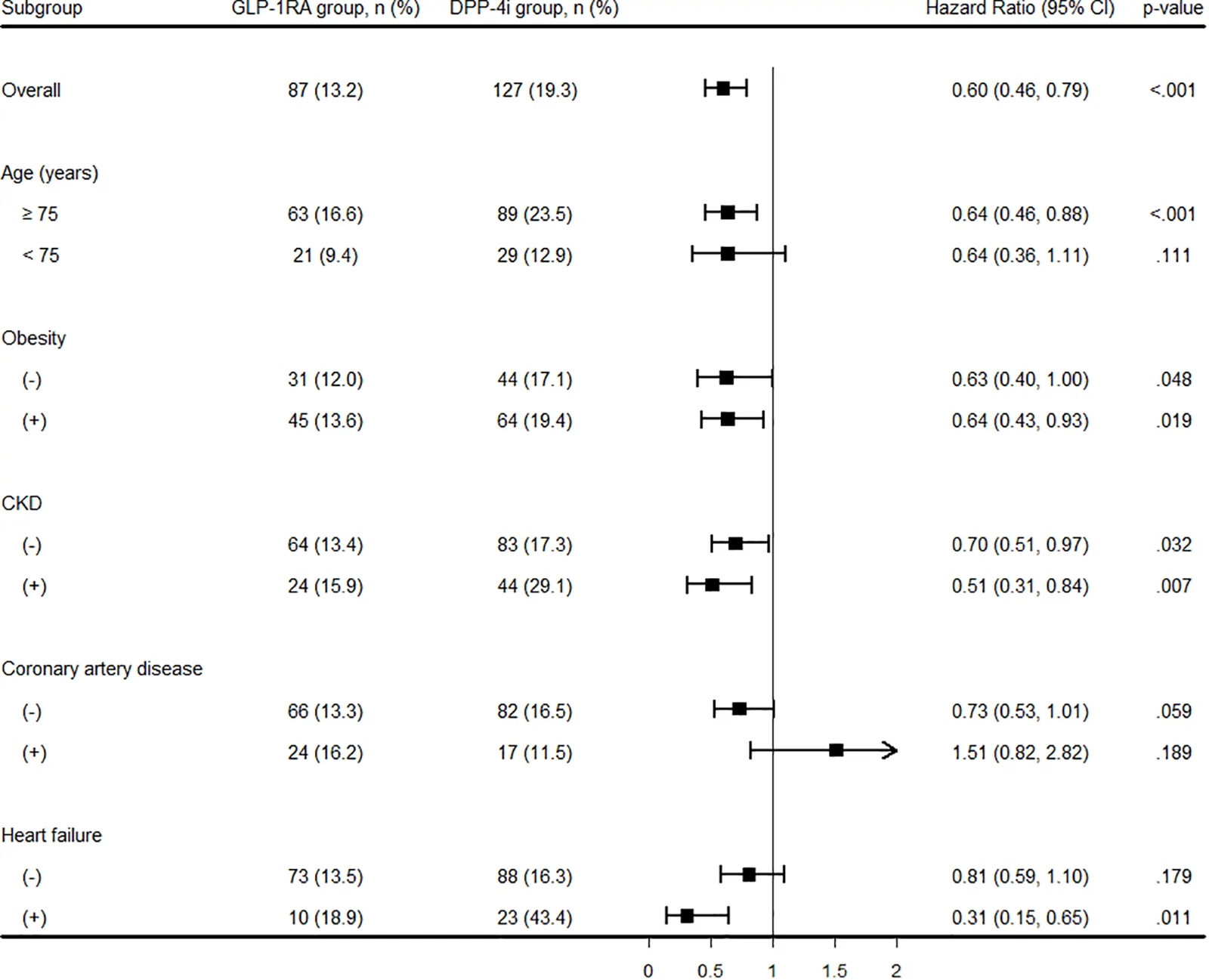
Forest plot of all-cause mortality with dipeptidyl peptidase-4 inhibitors (DPP-4i) comparator.

Compared with DPP-4i, the GLP-1RA group was associated with a significantly lower risk of all-cause mortality (27.5 vs. 40.4 events per 1,000 person-years; HR, 0.60; 95% CI, 0.46–0.79; **Table 2**). The corresponding E-value was 2.72 (95% LCL, 1.85). Kaplan–Meier time-to-event analysis demonstrated a significantly lower cumulative incidence of all-cause mortality in the GLP-1RA group compared with the DPP-4i group (log-rank p = 0.002; **Figure 2A**). Further stratified analysis demonstrated consistent trends, with significant differences across most subgroups (**Figure 3**).

**Table 2:** Hazard ratio of outcome between glucagon-like peptide-1 receptor agonists (GLP-1RAs) and their comparator groups.

Comparators	GLP-1RAs* events (IR/1000 PYs)	Comparator events (IR/1000 PYs)	HR (95% CI)	*P-*value	E-value (95% LCL)
DPP-4i (N = 659)					
Primary outcome: All-cause mortality	87 (27.5)	127 (40.4)	0.60 (0.46, 0.79)	0.0002	2.72 (1.85)
Secondary outcome					
MACEs	73 (29.7)	82 (30.6)	0.87 (0.63, 1.12)	0.3964	1.56 (1.00)
MAKEs	97 (32.4)	135 (45.4)	0.63 (0.48, 0.81)	0.0004	2.55 (1.77)
Thrombotic events	23 (7.4)	38 (12.1)	0.53 (0.32, 0.89)	0.0149	3.18 (1.50)
SGLT2i (N = 1,009)					
Primary outcome: All-cause mortality	103 (21.5)	130 (27.3)	0.76 (0.59, 0.99)	0.0390	1.96 (1.11)
Secondary outcome					
MACEs	102 (25.1)	90 (22.5)	1.12 (0.84, 1.49)	0.4381	1.49 (1.00)
MAKEs	118 (23.7)	136 (27.2)	0.87 (0.68, 1.11)	0.2481	1.56 (1.00)
Thrombotic events	40 (8.1)	31 (6.9)	1.13 (0.72, 1.79)	0.5893	1.51 (1.00)

IR, incidence rate; PYs, person-years; CI, confidence interval; DPP-4i, dipeptidyl peptidase 4 inhibitor; HR, hazard ratio; LCL, lower confidence limit; MACEs, major adverse cardiovascular events; MAKEs, major adverse kidney events; SGLT2i, sodium-glucose cotransporter 2 inhibitor.

*Propensity score matching (1:1 ratio) was performed separately for each comparison between the GLP-1RAs group and its respective comparator.

#### GLP-1RA vs. SGLT2i cohort

**Figure 4:**
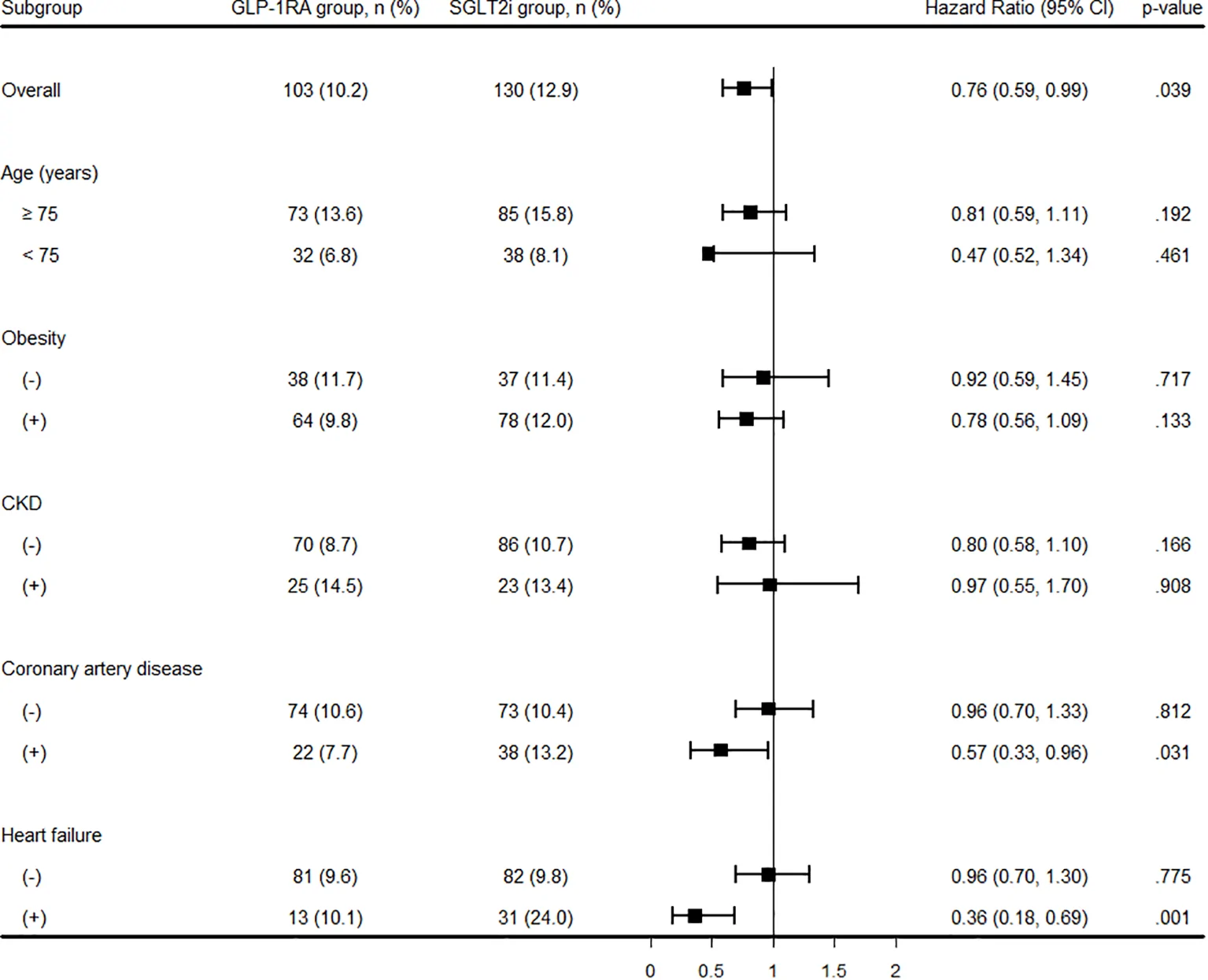
Forest plot of all-cause mortality with sodium-glucose cotransporter-2 inhibitors (SGLT2i) comparator.

In the comparison with SGLT2i, GLP-1RA use was associated with a significantly lower risk of all-cause mortality (21.5 vs. 27.3 events per 1,000 person-years; HR, 0.76; 95% CI, 0.59–0.99; **Table 2**). The corresponding E-value was 1.96 (95% LCL, 1.11). Kaplan–Meier survival analysis demonstrated a significantly lower cumulative incidence of all-cause mortality in the GLP-1RA group compared with the SGLT2i group (log-rank p = 0.039; **Figure 2B**). Results from stratified analyses demonstrated consistent findings, with significant differences in most subgroups (**Figure 4**).

### Secondary outcomes

Regarding secondary outcomes, in the comparison with DPP-4i, GLP-1RA use was associated with a significantly lower risk of MAKEs (HR, 0.63; 95% CI, 0.48–0.81) and thrombotic events (HR, 0.53; 95% CI, 0.32–0.89), whereas no significant difference was observed for MACEs (HR, 0.87; 95% CI, 0.63–1.12).

In the comparison with SGLT2i, no statistically significant differences were observed between GLP-1RA and SGLT2i for MACEs (HR, 1.12; 95% CI, 0.84–1.49), MAKEs (HR, 0.87; 95% CI, 0.68–1.11), or thrombotic events (HR, 1.13; 95% CI, 0.72–1.79).

## Discussion

In this large real-world cohort of patients with prostate cancer undergoing androgen deprivation therapy (ADT), use of glucagon-like peptide-1 receptor agonists (GLP-1RAs) was associated with a significantly lower risk of all-cause mortality compared with both DPP-4i and sodium–glucose cotransporter-2 inhibitors (SGLT2i). The magnitude of reduction was substantial versus DPP-4i (hazard ratio, 0.60; 95% CI, 0.46–0.79), corresponding to a 40% relative risk reduction, and remained significant though more modest versus SGLT2i (hazard ratio, 0.76; 95% CI, 0.59–0.99). GLP-1RA therapy was also associated with lower risks of major adverse kidney events and thrombotic complications compared with DPP-4i, whereas no significant difference was observed for major adverse cardiovascular events (MACEs). The elevated E-values for the primary and key secondary outcomes indicate that a strong unmeasured confounder would be required to fully account for the observed associations.

ADT is known to induce metabolic and vascular disturbances. Observational data have shown that ADT use is associated with up to an 84% higher risk of venous thromboembolism compared with non-use (28–31). Approximately 20% of patients experience MACEs after ADT initiation, contributing to increased hospitalization and mortality (6–8, 32). ADT has also been linked to acute kidney injury, potentially mediated by testosterone depletion and adverse effects on renal tubular function (33–36). These data underscore the need for careful cardiometabolic risk management in men receiving ADT.

GLP-1RAs have demonstrated consistent cardiovascular benefit in randomized trials. In SUSTAIN-6, semaglutide reduced MACEs by 26%, primarily driven by a reduction in stroke (37–39). In HARMONY, albiglutide reduced MACEs by 22% in patients with established cardiovascular disease (40). In REWIND, dulaglutide reduced MACEs by 12% in a population largely without prior cardiovascular disease, supporting its role in primary prevention (41, 42). More recently, the SELECT trial showed a 20% reduction in MACEs among obese individuals without diabetes but with established cardiovascular disease (43–45). Collectively, these trials support the broad cardiometabolic effects of GLP-1RAs beyond glycemic control.

Our findings extend this evidence to a high-risk oncologic population exposed to ADT. The reduction in mortality, kidney events, and thrombotic complications observed versus DPP-4i—a drug class with well-documented neutral cardiovascular effects (19–25, 46–53)—suggests that GLP-1RAs may effectively mitigate the adverse metabolic and vascular consequences of androgen suppression. The absence of a significant difference in MACEs may reflect limited statistical power, competing risks in this older population, or heterogeneity in cardiovascular phenotypes. The mortality benefit may be mediated through mechanisms beyond classical atherosclerotic events, including improved metabolic regulation, enhanced endothelial function, renal protection, and attenuation of pro-thrombotic states.

The comparison with SGLT2i also warrants careful interpretation. SGLT2i have consistently demonstrated substantial benefits in reducing hospitalization for heart failure and slowing the progression of chronic kidney disease in large randomized clinical trials and remain a preferred therapy for many patients with these conditions. In the present study, GLP-1RA use was associated with a modest reduction in all-cause mortality compared with SGLT2i, whereas no statistically significant differences were observed for MACEs, MAKEs, or thrombotic events. These neutral secondary findings should be interpreted cautiously because the relatively limited number of events may have reduced statistical power to detect modest between-group differences. Rather than indicating overall superiority of GLP-1RAs, our findings suggest that the two drug classes provide broadly comparable cardiometabolic protection, while GLP-1RAs may confer an additional mortality benefit in men with prostate cancer receiving ADT. Accordingly, SGLT2i remain an important therapeutic option, particularly for patients with heart failure or chronic kidney disease, and the present findings should be viewed as complementary rather than competitive evidence.

Emerging evidence also suggests potential cancer-related effects of GLP-1RAs. Observational studies have reported lower all-cause mortality among cancer survivors treated with GLP-1RAs (54). Experimental data indicate that GLP-1 receptor activation can inhibit tumor growth and promote apoptosis in colorectal and pancreatic cancer models, while in prostate cancer specifically, increased GLP-1 receptor expression has been observed in tumor tissue, and modulation of the PI3K/Akt pathway has been implicated in anti-proliferative effects (55). Furthermore, meta-analyses have reported a reduced incidence of obesity-related and prostate cancers among GLP-1RA users (56, 57). Although causal inference cannot be established from our findings, the observed survival benefit raises the possibility that GLP-1RAs may exert combined metabolic, vascular, renal, thrombotic, and potentially tumor-modifying effects.

The biological plausibility of these findings is supported by established mechanisms. GLP-1RAs improve insulin sensitivity, reduce visceral adiposity, decrease systemic inflammation, and enhance endothelial function (13, 58–60). In contrast, ADT promotes insulin resistance, central obesity, dyslipidemia, and pro-thrombotic states. By counteracting these pathways, GLP-1RAs may attenuate the adverse metabolic cascade induced by androgen suppression. The reduction in thrombotic events observed versus DPP-4i further supports a vascular protective effect.

Several methodological considerations regarding the follow-up definition warrant discussion. Follow-up began 30 days after the index date using a landmark approach to reduce reverse causation and minimize the influence of clinical events occurring immediately after treatment initiation, including events related to the underlying condition prompting treatment or early treatment modification. Although this approach is commonly adopted in pharmacoepidemiologic studies evaluating medication effectiveness, it necessarily excludes patients who died or experienced study outcomes during the landmark period and therefore may introduce survivor bias. Because the same landmark definition was applied to both treatment groups within each comparison, any resulting bias would likely affect both groups similarly. Nevertheless, the possibility that the landmark design influenced the observed associations cannot be completely excluded and should be considered when interpreting the findings.

This study has several strengths, including the use of a large multinational electronic health record database and rigorous propensity score matching with excellent covariate balance, as indicated by standardized mean differences below 0.1. Separate matching for each comparator minimized cross-class selection bias. The use of E-value analysis enhances the robustness of the findings.

Several limitations should be acknowledged. First, and most importantly, detailed oncological characteristics, including tumor stage, Gleason score, metastatic status, and duration of ADT, were unavailable within the TriNetX database and therefore could not be incorporated into the propensity score model. Because these factors may influence both treatment selection and mortality risk, residual confounding cannot be excluded. The direction of this potential bias is uncertain. If patients receiving GLP-1RAs had less advanced disease at baseline, the observed mortality benefit may have been overestimated. Conversely, if GLP-1RAs were preferentially prescribed to patients with greater metabolic risk or avoided in frailer patients with advanced disease, the observed association may have underestimated the true treatment effect.

Second, information regarding diabetes duration, medication adherence, and cause-specific mortality was unavailable, limiting mechanistic interpretation of the observed associations. Third, the relatively small number of events for several secondary outcomes, particularly in the GLP-1RA versus SGLT2i comparison, may have limited statistical power to detect modest between-group differences. Fourth, because all-cause rather than prostate cancer-specific mortality was evaluated, the relative contributions of cardiovascular, metabolic, and oncologic mechanisms could not be distinguished. Fifth, although the 30-day landmark design was intended to reduce reverse causation, exclusion of early post-index events may have introduced survivor bias. Finally, as with any observational study using routinely collected electronic health records, residual confounding and limited generalizability to populations outside the TriNetX network remain possible.

In conclusion, among patients with prostate cancer receiving ADT, GLP-1RA therapy was associated with lower all-cause mortality compared with DPP-4i and SGLT2i, as well as lower risks of kidney and thrombotic events versus DPP-4i. These findings support consideration of GLP-1RAs as a glucose-lowering strategy in this metabolically and vascularly vulnerable population. Prospective studies are needed to confirm these associations and clarify underlying mechanisms.

## Conclusions

In summary, GLP-1RA therapy was associated with lower all-cause mortality than DPP-4i and SGLT2i among men with prostate cancer receiving ADT and concomitant T2D. Additional reductions in kidney and thrombotic events were observed compared with DPP-4i. No statistically significant differences were observed between GLP-1RA and SGLT2i for cardiovascular, kidney, or thrombotic outcomes; however, these neutral findings should be interpreted cautiously because the limited number of events may have reduced statistical power to detect modest between-group differences. These findings suggest that GLP-1RAs may represent a favorable therapeutic option in this metabolically and vascularly vulnerable population. Prospective studies are needed to confirm these associations and clarify the underlying mechanisms.

## Data Availability

The raw data supporting the conclusions of this article will be made available by the authors, without undue reservation.
