# Supplementary material for: Glucagon-like peptide-1 receptor agonist versus other anti-diabetic agents on patients with prostate cancer undergoing androgen-deprivation therapy
Source: Front Urol. 2026 Aug 7;6:1855180. doi: 10.3389/fruro.2026.1855180 (PMC13494580; doi:10.3389/fruro.2026.1855180)
Supplement: Supplementary file 1 [file Table1.docx]

**Table S1.** Demographic, diagnostic, procedural, medication, visit, and laboratory codes utilized in the definition of the cohorts.

| **Category** | **Code** | **Description** |
| --- | --- | --- |
| **Glucagon-like peptide-1 analogues** | | |
| medication | NLM:ATC:A10BJ | Glucagon-like peptide-1 (GLP-1) analogues |
| medication | NLM:RXNORM:1991302 | semaglutide |
| medication | NLM:RXNORM:475968 | liraglutide |
| medication | NLM:RXNORM:2601723 | tirzepatide |
| medication | NLM:RXNORM:1551291 | dulaglutide |
| Sodium-glucose co-transporter 2 inhibitors | | |
| medication | NLM:ATC:A10BK | Sodium-glucose co-transporter 2 (SGLT2) inhibitors |
| Dipeptidyl peptidase 4 (DPP-4) inhibitors | | |
| medication | NLM:ATC:A10BH | Dipeptidyl peptidase 4 (DPP-4) inhibitors |
| **Prostate cancer with Type 2 diabetes mellitus** | | |
| diagnosis | UMLS:ICD10CM:C61 | Malignant neoplasm of prostate |
| globaloncology | UMLS:ICDO3:C61 | PROSTATE GLAND |
| diagnosis | UMLS:ICD10CM:E11 | Type 2 diabetes mellitus |
| **Androgen lowering treatments** | | |
| procedure | UMLS:CPT:1008565 | Orchiectomy, radical, for tumor |
| procedure | UMLS:ICD10PCS:0VB9 | Male Reproductive System / Excision / Testis, Right |
| procedure | UMLS:ICD10PCS:0VBB | Male Reproductive System / Excision / Testis, Left |
| procedure | UMLS:ICD10PCS:0VBC | Male Reproductive System / Excision / Testes, Bilateral |
| medication | NLM:RXNORM:42375 | leuprolide |
| medication | NLM:RXNORM:2472778 | relugolix |
| medication | NLM:RXNORM:50610 | goserelin |
| medication | NLM:RXNORM:38782 | triptorelin |
| medication | NLM:RXNORM:50975 | histrelin |
| procedure | UMLS:CPT:54520 | Orchiectomy, simple (including subcapsular), with or without testicular prosthesis, scrotal or inguinal approach |
| procedure | UMLS:CPT:54522 | Orchiectomy, partial |
| medication | NLM:RXNORM:475230 | degarelix |
| medication | NLM:RXNORM:1100072 | abiraterone |
| medication | NLM:RXNORM:1999574 | apalutamide |
| medication | NLM:RXNORM:2180325 | darolutamide |
| medication | NLM:RXNORM:1307298 | enzalutamide |
| **Exclusion criteria** | | |
| diagnosis | UMLS:ICD10CM:I21 | Acute myocardial infarction |
| diagnosis | UMLS:ICD10CM:I22 | Subsequent ST elevation (STEMI) and non-ST elevation (NSTEMI) myocardial infarction |
| diagnosis | UMLS:ICD10CM:I62 | Other and unspecified nontraumatic intracranial hemorrhage |
| diagnosis | UMLS:ICD10CM:I61 | Nontraumatic intracerebral hemorrhage |
| diagnosis | UMLS:ICD10CM:I63 | Cerebral infarction |
| diagnosis | UMLS:ICD10CM:I46 | Cardiac arrest |
| diagnosis | UMLS:ICD10CM:I46.9 | Cardiac arrest, cause unspecified |
| diagnosis | UMLS:ICD10CM:I21 | Acute myocardial infarction |
| diagnosis | UMLS:ICD10CM:I22 | Subsequent ST elevation (STEMI) and non-ST elevation (NSTEMI) myocardial infarction |
| diagnosis | UMLS:ICD10CM:I62 | Other and unspecified nontraumatic intracranial hemorrhage |
| diagnosis | UMLS:ICD10CM:I61 | Nontraumatic intracerebral hemorrhage |
| diagnosis | UMLS:ICD10CM:I63 | Cerebral infarction |
| diagnosis | UMLS:ICD10CM:I46 | Cardiac arrest |
| diagnosis | UMLS:ICD10CM:I46.9 | Cardiac arrest, cause unspecified |
| diagnosis | UMLS:ICD10CM:N18.5 | Chronic kidney disease, stage 5 |
| diagnosis | UMLS:ICD10CM:N18.6 | End stage renal disease |
| diagnosis | UMLS:ICD10CM:Z99.2 | Dependence on renal dialysis |
| diagnosis | UMLS:ICD10CM:N19 | Unspecified kidney failure |
| diagnosis | UMLS:ICD10CM:R99 | Ill-defined and unknown cause of mortality |
| procedure | UMLS:ICD9CM:39.95 | Hemodialysis |
| procedure | UMLS:CPT:1012740 | Dialysis Services and Procedures |
| procedure | UMLS:CPT:1029674 | Dialysis Circuit Procedures |
| demographics | Deceased | Deceased |
| laboratory | TNX:8001 | Glomerular filtration rate/1.73 sq M.predicted [Volume Rate/Area] in Serum, Plasma or Blood by Creatinine-based formula (MDRD) (at most 5.00 mL/min/{1.73_m2}) |
| diagnosis | UMLS:ICD10CM:N18.5 | Chronic kidney disease, stage 5 |
| diagnosis | UMLS:ICD10CM:N18.6 | End stage renal disease |
| diagnosis | UMLS:ICD10CM:Z99.2 | Dependence on renal dialysis |
| diagnosis | UMLS:ICD10CM:N19 | Unspecified kidney failure |
| diagnosis | UMLS:ICD10CM:R99 | Ill-defined and unknown cause of mortality |
| procedure | UMLS:ICD9CM:39.95 | Hemodialysis |
| procedure | UMLS:CPT:1012740 | Dialysis Services and Procedures |
| procedure | UMLS:CPT:1029674 | Dialysis Circuit Procedures |
| demographics | Deceased | Deceased |
| laboratory | TNX:8001 | Glomerular filtration rate/1.73 sq M.predicted [Volume Rate/Area] in Serum, Plasma or Blood by Creatinine-based formula (MDRD) (at most 5.00 mL/min/{1.73_m2}) |
| diagnosis | UMLS:ICD10CM:I26 | Pulmonary embolism |
| diagnosis | UMLS:ICD10CM:I82.40 | Acute embolism and thrombosis of unspecified deep veins of lower extremity |
| diagnosis | UMLS:ICD10CM:I82.409 | Acute embolism and thrombosis of unspecified deep veins of unspecified lower extremity |
| diagnosis | UMLS:ICD10CM:I82 | Other venous embolism and thrombosis |

**Table S2.** Definitions of covariates coding used in this study.

| **Code** | **Description** |
| --- | --- |
| Age | Current Age |
| AI | Age at Index |
| F | Female |
| 2054-5 | Black or African American |
| M | Male |
| 2106-3 | White |
| 1002-5 | American Indian or Alaska Native |
| UNK | Unknown Race |
| 2076-8 | Native Hawaiian or Other Pacific Islander |
| UN | Unknown Ethnicity |
| 2186-5 | Not Hispanic or Latino |
| 2135-2 | Hispanic or Latino |
| 2131-1 | Other Race |
| 2028-9 | Asian |
| I50 | Heart failure |
| I48 | Atrial fibrillation and flutter |
| I60-I69 | Cerebrovascular diseases |
| I73.9 | Peripheral vascular disease, unspecified |
| N18 | Chronic kidney disease (CKD) |
| K74 | Fibrosis and cirrhosis of liver |
| K75 | Other inflammatory liver diseases |
| M30-M36 | Systemic connective tissue disorders |
| M05 | Rheumatoid arthritis with rheumatoid factor |
| M10 | Gout |
| I50 | Heart failure |
| I48 | Atrial fibrillation and flutter |
| I60-I69 | Cerebrovascular diseases |
| CV800 | ACE INHIBITORS |
| CV100 | BETA BLOCKERS/RELATED |
| CV200 | CALCIUM CHANNEL BLOCKERS |
| CV700 | DIURETICS |
| CV300 | ANTIARRHYTHMICS |
| CV350 | ANTILIPEMIC AGENTS |
| A10BA | Biguanides |
| A10BB | Sulfonylureas |
| A10BK | Sodium-glucose co-transporter 2 (SGLT2) inhibitors |
| A10BH | Dipeptidyl peptidase 4 (DPP-4) inhibitors |
| A10BG | Thiazolidinediones |
| A10A | INSULINS AND ANALOGUES |
| 9057 | Prostate specific Ag [Mass/volume] in Serum or Plasma, 2 - 0 ng/mL |
| 9083 | BMI, 30 - 0 kg/m2 |
| 9037 | Hemoglobin A1c/Hemoglobin.total in Blood, 9 - 0 % |
| 9014 | Hemoglobin [Mass/volume] in Blood |
| 9085 | Blood Pressure, Systolic |
| 9002 | Cholesterol in LDL [Mass/volume] in Serum or Plasma |
| 9000 | Cholesterol [Mass/volume] in Serum or Plasma |
| 8001 | Glomerular filtration rate/1.73 sq M.predicted [Volume Rate/Area] in Serum, Plasma or Blood by Creatinine-based formula (MDRD), 0 - 60 mL/min/{1.73_m2} |
| 39156-5 | Body Mass Index |
| 9057 | Prostate specific Ag [Mass/volume] in Serum or Plasma |

**Table S3.** Definitions of outcomes coding used in this study.

| **Code** | **Description** |
| --- | --- |
| **1#: All-cause mortality** | |
| Deceased | Deceased |
| UMLS:ICD10CM:R99 | Ill-defined and unknown cause of mortality |
| **2#: MACEs** | |
| UMLS:ICD10CM:I61 | Nontraumatic intracerebral hemorrhage |
| UMLS:ICD10CM:I62 | Other and unspecified nontraumatic intracranial hemorrhage |
| UMLS:ICD10CM:I63 | Cerebral infarction |
| UMLS:ICD10CM:I65 | Occlusion and stenosis of precerebral arteries, not resulting in cerebral infarction |
| UMLS:ICD10CM:I66 | Occlusion and stenosis of cerebral arteries, not resulting in cerebral infarction |
| UMLS:ICD10CM:I67.89 | Other cerebrovascular disease |
| UMLS:ICD10CM:I50.21 | Acute systolic (congestive) heart failure |
| UMLS:ICD10CM:I50.23 | Acute on chronic systolic (congestive) heart failure |
| UMLS:ICD10CM:I50.31 | Acute diastolic (congestive) heart failure |
| UMLS:ICD10CM:I50.33 | Acute on chronic diastolic (congestive) heart failure |
| UMLS:ICD10CM:I50.41 | Acute combined systolic (congestive) and diastolic (congestive) heart failure |
| UMLS:ICD10CM:I50.43 | Acute on chronic combined systolic (congestive) and diastolic (congestive) heart failure |
| UMLS:ICD10CM:I46.2 | Cardiac arrest due to underlying cardiac condition |
| UMLS:ICD10CM:I46.9 | Cardiac arrest, cause unspecified |
| UMLS:ICD10CM:I47.0 | Re-entry ventricular arrhythmia |
| UMLS:ICD10CM:I47.2 | Ventricular tachycardia |
| UMLS:ICD10CM:I49.0 | Ventricular fibrillation and flutter |
| UMLS:ICD10CM:I49.3 | Ventricular premature depolarization |
| **3#: MAKES** | |
| UMLS:ICD10CM:N18.5 | Chronic kidney disease, stage 5 |
| UMLS:ICD10CM:N18.6 | End stage renal disease |
| UMLS:ICD10CM:Z99.2 | Dependence on renal dialysis |
| UMLS:ICD10CM:N19 | Unspecified kidney failure |
| UMLS:ICD10CM:Z99.2 | Dependence on renal dialysis |
| UMLS:ICD10CM:R99 | Ill-defined and unknown cause of mortality |
| UMLS:ICD9CM:39.95 | Hemodialysis |
| UMLS:CPT:1012740 | Dialysis Services and Procedures |
| UMLS:CPT:1029674 | Dialysis Circuit Procedures |
| Deceased | Deceased |
| TNX:8001 | Glomerular filtration rate/1.73 sq M.predicted [Volume Rate/Area] in Serum, Plasma or Blood by Creatinine-based formula (MDRD) (Data Source: TriNetX; at most 5.00 mL/min/{1.73_m2} (most recent occurrence)) |
| **4#: Thrombotic events** | |
| UMLS:ICD10CM:I26 | Pulmonary embolism |
| UMLS:ICD10CM:I82.40 | Acute embolism and thrombosis of unspecified deep veins of lower extremity |
| UMLS:ICD10CM:I82 | Other venous embolism and thrombosis |
| UMLS:ICD10CM:I82.409 | Acute embolism and thrombosis of unspecified deep veins of unspecified lower extremity |

ICD10CM, International Classification of Diseases, Tenth Revision, Clinical Modification

NLM, National Library of Medicine

RXNORM, medical prescription normalized

UMLS, Unified Medical Language System

**Table S4**. Pre-matched baseline characteristics of glucagon-like peptide-1 receptor agonists (GLP-1RAs) and their comparator cohorts.

|  |  | **GLP-1RAs** | | **DPP4i** | **SMDs** | |  | **GLP-1RAs** | **SGLT2i** | | **SMDs** | |
| --- | --- | --- | --- | --- | --- | --- | --- | --- | --- | --- | --- | --- |
|  |  | n=1,366 | n=1,587 | | |  |  | n=1.366 | | n=1,755 | |  |
| **Age at Index (mean ± SD)** |  | 69.9 ± 8 | | 74.5 ± 8.2 | 0.568 | |  | 69.9 ± 8 | 73.7 ± 8.1 | | 0.472 | |
| **Ethnicity (%)** |  |  | |  |  | |  |  |  | |  | |
| White |  | 881 (64.5) | | 664 (41.8) | 0.466 | |  | 881 (64.5) | 995 (56.7) | | 0.16 | |
| Black or African American |  | 352 (25.8) | | 276 (17.4) | 0.205 | |  | 352 (25.8) | 410 (23.4) | | 0.056 | |
| Asian |  | 33 (2.4) | | 249 (15.7) | 0.476 | |  | 33 (2.4) | 118 (6.7) | | 0.207 | |
| Unknown Ethnicity |  | 251 (18.4) | | 513 (32.3) | 0.325 | |  | 251 (18.4) | 388 (22.1) | | 0.093 | |
| **Race (%)** |  |  | |  |  | |  |  |  | |  | |
| Not Hispanic or Latino |  | 1,027 (75.2) | | 994 (62.6) | 0.274 | |  | 1,027 (75.2) | 1,273 (72.5) | | 0.06 | |
| Hispanic or Latino |  | 88 (6.4) | | 80 (5) | 0.06 | |  | 88 (6.4) | 94 (5.4) | | 0.046 | |
| Other Race |  | 47 (3.4) | | 39 (2.5) | 0.058 | |  | 47 (3.4) | 60 (3.4) | | 0.001 | |
| Unknown Race |  | 45 (3.3) | | 346 (21.8) | 0.582 | |  | 45 (3.3) | 157 (8.9) | | 0.237 | |
| **Substance use (%)** |  |  | |  |  | |  |  |  | |  | |
| Tobacco use |  | 27 (2) | | 21 (1.3) | 0.051 | |  | 27 (2) | 43 (2.5) | | 0.032 | |
| Nicotine dependence |  | 110 (8.1) | | 114 (7.2) | 0.033 | |  | 110 (8.1) | 182 (10.4) | | 0.08 | |
| Alcohol related disorders |  | 19 (1.4) | | 18 (1.1) | 0.023 | |  | 19 (1.4) | 37 (2.1) | | 0.055 | |
| **Comorbidities (%)** |  |  | |  |  | |  |  |  | |  | |
| Type 2 diabetes mellitus |  | 1,250 (91.5) | | 1,343 (84.6) | 0.214 | |  | 1,250 (91.5) | 1,610 (91.7) | | 0.008 | |
| Hyperlipidemia |  | 601 (44) | | 560 (35.3) | 0.179 | |  | 601 (44) | 814 (46.4) | | 0.048 | |
| Essential hypertension |  | 1,029 (75.3) | | 1,009 (63.6) | 0.257 | |  | 1,029 (75.3) | 1,310 (74.6) | | 0.016 | |
| Chronic kidney disease |  | 271 (19.8) | | 366 (23.1) | 0.079 | |  | 271 (19.8) | 555 (31.6) | | 0.272 | |
| Atrial fibrillation and flutter |  | 152 (11.1) | | 160 (10.1) | 0.034 | |  | 152 (11.1) | 382 (21.8) | | 0.29 | |
| Heart failure |  | 137 (10) | | 166 (10.5) | 0.014 | |  | 137 (10) | 490 (27.9) | | 0.469 | |
| Cerebrovascular diseases |  | 98 (7.2) | | 132 (8.3) | 0.043 | |  | 98 (7.2) | 161 (9.2) | | 0.073 | |
| Peripheral vascular disease |  | 61 (4.5) | | 64 (4) | 0.021 | |  | 61 (4.5) | 129 (7.4) | | 0.123 | |
| Gout |  | 80 (5.9) | | 99 (6.2) | 0.016 | |  | 80 (5.9) | 149 (8.5) | | 0.102 | |
| **Medications (%)** |  |  | |  |  | |  |  |  | |  | |
| *Antihypertensive Agents* |  |  | |  |  | |  |  |  | |  | |
| ACE inhibitors |  | 311 (22.8) | | 307 (19.3) | 0.084 | |  | 311 (22.8) | 391 (22.3) | | 0.012 | |
| Beta blockers/related |  | 442 (32.4) | | 483 (30.4) | 0.041 | |  | 442 (32.4) | 754 (43) | | 0.22 | |
| Diuretics |  | 439 (32.1) | | 458 (28.9) | 0.071 | |  | 439 (32.1) | 712 (40.6) | | 0.176 | |
| Calcium channel blockers |  | 384 (28.1) | | 474 (29.9) | 0.039 | |  | 384 (28.1) | 529 (30.1) | | 0.045 | |
| *Antidiabetic Agents* |  |  | |  |  | |  |  |  | |  | |
| Biguanides |  | 562 (41.1) | | 587 (37) | 0.085 | |  | 562 (41.1) | 613 (34.9) | | 0.128 | |
| Insulins and analogues |  | 444 (32.5) | | 500 (31.5) | 0.021 | |  | 444 (32.5) | 581 (33.1) | | 0.013 | |
| Sulfonylureas |  | 235 (17.2) | | 327 (20.6) | 0.087 | |  | 235 (17.2) | 243 (13.8) | | 0.093 | |
| Thiazolidinediones |  | 38 (2.8) | | 37 (2.3) | 0.029 | |  | 38 (2.8) | 37 (2.1) | | 0.044 | |
| Antilipemic agents |  | 695 (50.9) | | 708 (44.6) | 0.126 | |  | 695 (50.9) | 962 (54.8) | | 0.079 | |
| Antiarrhythmics |  | 518 (37.9) | | 451 (28.4) | 0.203 | |  | 518 (37.9) | 721 (41.1) | | 0.065 | |
| **Lab (%)** |  |  | |  |  | |  |  |  | |  | |
| BMI ≥ 30 kg/m² |  | 910 (66.6) | | 433 (27.3) | 0.858 | |  | 597 (59.2) | 599 (59.4) | | 0.004 | |
| HbA1c ≥ 9 % |  | 319 (23.4) | | 264 (16.6) | 0.169 | |  | 319 (23.4) | 260 (14.8) | | 0.219 | |
| eGFR ≤ 60 (mL/min/1.73 m²) |  | 443 (32.4) | | 648 (40.8) | 0.175 | |  | 443 (32.4) | 805 (45.9) | | 0.278 | |
| PSA ≥0.1 (ng/mL) |  | 609 (44.6) | | 663 (41.8) | 0.057 | |  | 609 (44.6) | 792 (45.1) | | 0.011 | |
| PSA ≥2.0 (ng/mL) |  | 335 (24.5) | | 445 (28) | 0.08 | |  | 335 (24.5) | 464 (26.4) | | 0.044 | |

DPP4i, dipeptidyl peptidase 4 inhibitor; SGLT2i, sodium-glucose cotransporter 2 inhibitor; SMDs, standardized mean difference; SD, standard deviation; BMI, body mass index (kg/m^2^); eGFR, estimated glomerular filtration rate (mL/min/1.73 m²); HbA1c, glycated hemoglobin.(%); PSA, prostate-specific antigen (ng/mL).
